# Cardiac Arrest Disrupts Caspase-1 and Patterns of Inflammatory Mediators Differently in Skin and Muscle Following Localized Tissue Injury in Rats: Insights from Data-Driven Modeling

**DOI:** 10.3389/fimmu.2015.00587

**Published:** 2015-11-20

**Authors:** Ravi Starzl, Dolores Wolfram, Ruben Zamora, Bahiyyah Jefferson, Derek Barclay, Chien Ho, Vijay Gorantla, Gerald Brandacher, Stefan Schneeberger, W. P. Andrew Lee, Jaime Carbonell, Yoram Vodovotz

**Affiliations:** ^1^Language Technologies Institute, Carnegie Mellon University, Pittsburgh, PA, USA; ^2^Department of Plastic and Reconstructive Surgery, University of Pittsburgh, Pittsburgh, PA, USA; ^3^Department of Plastic and Reconstructive Surgery, Johns Hopkins Medicine, Baltimore, MD, USA; ^4^Department of Plastic and Reconstructive Surgery, Innsbruck Medical University, Innsbruck, Austria; ^5^Department of Surgery, University of Pittsburgh, Pittsburgh, PA, USA; ^6^Center for Inflammation and Regenerative Modeling, McGowan Institute for Regenerative Medicine, University of Pittsburgh, Pittsburgh, PA, USA; ^7^Department of Biological Sciences, Carnegie Mellon University, Pittsburgh, PA, USA

**Keywords:** Inflammasome, inflammatory mediators, computational modeling, data driven modeling, cardiac arrest and trauma, immunoregulatory, localized tissue injury, hemorrhagic shock

## Abstract

**Background:**

Trauma often cooccurs with cardiac arrest and hemorrhagic shock. Skin and muscle injuries often lead to significant inflammation in the affected tissue. The primary mechanism by which inflammation is initiated, sustained, and terminated is cytokine-mediated immune signaling, but this signaling can be altered by cardiac arrest. The complexity and context sensitivity of immune signaling in general has stymied a clear understanding of these signaling dynamics.

**Methodology/principal findings:**

We hypothesized that advanced numerical and biological function analysis methods would help elucidate the inflammatory response to skin and muscle wounds in rats, both with and without concomitant shock. Based on the multiplexed analysis of inflammatory mediators, we discerned a differential interleukin (IL)-1α and IL-18 signature in skin vs. muscle, which was suggestive of inflammasome activation in the skin. Immunoblotting revealed caspase-1 activation in skin but not muscle. Notably, IL-1α and IL-18, along with caspase-1, were greatly elevated in the skin following cardiac arrest, consistent with differential inflammasome activation.

**Conclusion/significance:**

Tissue-specific activation of caspase-1 and the NLRP3 inflammasome appear to be key factors in determining the type and severity of the inflammatory response to tissue injury, especially in the presence of severe shock, as suggested via data-driven modeling.

## Introduction

Healthy skin and muscle tissue exist in a steady-state equilibrium that is characterized in large part by the absence of acute inflammation (1). When injured, the tissue’s steady state is upset, inducing a cascade of responses that include inflammation, repair, and remodeling of the affected region. The inflammatory response is an essential part of successful wound healing and sets the stage for effective repair and remodeling (2). The composition of the underlying inflammatory/immune signaling that drives the inflammatory response to injury is therefore critical in determining whether the inflammation ultimately leads to successful healing or instead leads to additional damage and dysfunctional tissue healing. However, when inflammatory signaling proportional to an injury is altered, the inflammatory response does not successfully transition to subsequent stages of tissue healing (3). Significant systemic insults, such as cardiac arrest, have the potential to drastically alter local immune signaling activity and possibly disrupt wound healing. To the best of our knowledge, the effect of cardiac arrest on these local wound-healing-associated immune signaling processes has not been elucidated.

In the setting of both civilian and military trauma, severe local tissue injury can often be accompanied by cardiac arrest (4–7). Cardiac arrest affects numerous physiological processes, as well as setting in motion systemic inflammation (8–11). Although short-term hypoxia may be an important part of stimulating wound healing (12), severe or extended disruption of oxygen supply interferes with successful wound healing (13). We hypothesize that the specific local inflammatory mediator network patterns expressed during the initial inflammatory response contain information about how the immune system is responding to a localized injury, and whether the response is leading to successful or dysfunctional healing outcome.

Cardiac arrest, even with eventual sudden restoration of blood flow, is known to cause significant cellular stress, the buildup of toxins, and the release of endogenous danger signals that can promote inflammation. In the neurological context, cardiac arrest is particularly well understood to cause a great deal of damage to nerves. Therefore, we further hypothesize that the eventual sudden restoration of blood flow will in essence create an ischemia/reperfusion milieu characterized by the further release of inflammatory mediators, which will have a further deleterious effect on properly localized and moderated wound healing.

The domain of wound healing has been extensively studied with many excellent articles and reviews that provide an overview of the phases, cellular processes, and molecular signals that have been observed in various wound-healing settings (2, 14–18). The effect of the insult of cardiac arrest on patient outcomes has been studied with respect to the impact on patient survival and neurological damage, with significant focus on the potential protective effects of induced mild hypothermia (19–22). However, to the best of our knowledge, no prior studies examine the effect that cardiac arrest may have on the local immune signaling processes essential to inflammation and wound healing.

Analysis and interpretation of the immune signaling process that drives inflammation is challenging because of the complex interplay among inflammatory mediators and their sensitive dependence on local and systemic conditions. Consequently, analysis of the progression of inflammation must leverage analytic methods and the insights that are capable of robustly assessing multiple dimensions of variance simultaneously, and of obtaining relevant information from high-dimensional data matrices (23).

A variety of data analysis methods suitable to exposing the internal structure of such noisy, high-dimensional data have been developed and utilized extensively for the analysis of complex systems in areas such as computational linguistics and machine learning (24). We and others have suggested the need to employ data-driven computational modeling in order to derive insights from the types of high-dimensional datasets obtained when studying complex biological system, such as the inflammatory response (25–27). We have explored multiple techniques for visualizing and enumerating salient features of acute inflammation in both preclinical and clinical settings, thus providing some insight into the types of analytic methods likely to be effective in elucidating key aspects of the observed inflammatory response (25, 28–37).

In the present study, we investigated patterns of immune signaling induced *in vivo* in rat skin and muscle following tissue injury in the form of excisional wounding. We then carried out *in silico* analyses to define principal drivers of local inflammatory responses, in the presence or absence of cardiac arrest. We demonstrate that tissue-specific immune signaling patterns are modified by cardiac arrest (also a paradigm of severe hemorrhagic shock) and suggest that inflammasome activity may govern the type of inflammation initiated.

## Materials and Methods

### Rat Model of Tissue Injury

To simulate tissue injury, we carried out deep tissue excisional biopsies of skin and muscle (38, 39). All animal procedures, care, and housing were reviewed and approved by the University of Pittsburgh Institutional Animal Care and Use Committee and followed the National Institutes of Health guidelines for the care and use of laboratory animals. We divided the study into two experimental groups: injury only (injury group) and injury with cardiac arrest (cardiac arrest group). In the “injury group,” four Lewis rats were anesthetized, and an excision biopsy was taken from the lateral aspect of the thigh on one of the hind limbs in each of the rats. Tissue was drawn away from the body and held in forceps while surgical scissors cut 15 mm × 10 mm of tissue from the lateral aspect of the thigh. In the “cardiac arrest group,” four Lewis rats were sacrificed with a fatal sodium pentobarbital (Lundbeck Inc., Deerfield, IL, USA) overdose, and excision biopsy taken 15–30 s after cessation of heartbeat.

### Protein Isolation and Sample Preparation

We have previously shown the preservation of animal and human tissues in RNALater™ (Ambion, Austin, TX, USA) is a method compatible with subsequent Luminex™ analysis (40–42). Accordingly, all tissue samples were sectioned into ≤0.5 cm^3^ pieces and placed into individual sample tubes filled with RNALater™ and stored as per manufacturer instructions and as determined empirically in our prior study (40). For tissue processing, approximately 50 mg of the tissue was transferred to a 2-ml microcentrifuge tube containing 0.6 ml of 1× BioSource™ (Invitrogen, San Diego, CA, USA) tissue extraction reagent supplemented with 10 μl of 100mM phenylmethanesulfonyl fluoride in ethanol as a protease inhibitor. The tissues were then homogenized using a tissue homogenizer, then centrifuged at 4°C for 10 min at 10,000 × *g*. After centrifugation, the supernatant were collected and assayed for protein content using the bicinchoninic acid (BCA) protein assay (Pierce, Rockford, IL, USA) as per manufacturer’s protocol.

### Assays for Inflammation Biomarkers

All samples were assayed for inflammatory cytokines and chemokines using the Luminex™ multiplexing platform (100 IS; MiraiBio, Alameda, CA, USA) and a Millipore™ 14-plex rat cytokine bead set (Millipore, Billerica, MA, USA) that included interferon (IFN) γ, interleukin (IL)-1α, IL-1β, IL-2, IL-4, IL-5, IL-6, IL-10, IL-12p70, IL-18, monocyte chemotactic protein (MCP-1), GRO/KC, TNFα, and granulocyte-macrophage colony-stimulating factor (GM-CSF). Results were read in picogram per milliliter, then subsequently normalized to total mass of sample protein (picogram of cytokine/milligram of protein) for each of the 14 cytokines by the formula *x* = (*a*/*b*) × 1000, where *x* = (picogram of cytokine/milligram of protein), *a* = (picogram per milliliter of cytokine), and *b* = (microgram per milliliter of protein).

For immunodetection of caspase-1, protein samples (25 μg) were separated on 12% SDS-polyacrylamide gels, and the gels were electroblotted onto PVDF membranes. After overnight blocking, the membranes were incubated overnight with a rabbit polyclonal antibody from Abcam (Cambridge, MA, USA) at 4°C followed by 1 h incubation with a goat antirabbit secondary antibody from Pierce (Rockford, IL, USA) at room temperature. Bands were detected using the Supersignal™ West Dura Extended Duration Substrate Chemiluminescent kit as per the manufacturer instructions. All readings are for active caspase-1 (20 kDa).

For Coomassie blue staining, the same procedure was followed as for acrylamide gels (4–15%), then stained with Bio-safe™ (BioRad, Hercules, CA, USA) stain. After washing, 50 ml of Bio-safe™ Coomassie stain was added. After shaking for 1 h, the protein bands became visible within 20 min and reached maximum intensity within 1 h. Skin to muscle caspase-1 expression was calculated by dividing the measured band relative density (BRD) ratio of skin with the measured BRD ratio of muscle: *r* = *s*/*m*, where *s* = skin BRD, *m* = muscle BRD, and *r* = ratio of skin BRD to muscle BRD. SEM was calculated for each group.

### Statistical and Computational Analyses

Statistical analyses were performed using Math Works MatLab™, Microsoft Excel™, and SAS Stat View™. Western blot quantifications were compared using a balanced one-way ANOVA with 5% significance. Cytokine quantifications were compared utilizing an unpaired one-tailed heteroscedastic *t*-test, again with a 5% significance level.

Principal component analysis (PCA) (26) was used to help discern which of the 14 proinflammatory cytokines and chemokines measured by Luminex™ are the most informative with regards to the observed immune response, similar to methodology we have used previously in the context of murine trauma/hemorrhage (25). Data were grouped by tissue (all skin samples and all muscle samples), and linear combinations of the original 14 dimensions (cytokines/chemokines) in each group were created in order to produce synthesized latent variables that explain >95% of the variance observed. The strength of each inflammatory mediator’s contribution to each of the principal components was also examined, in order to provide additional evidence for determining which cytokines/chemokines could be considered to be driving the observed immune reactions (25).

Immune signaling is driven to a large extent by the local inflammatory mediator milieu. While methods, such as PCA, are able to identify mediators that contribute the most variance to the observed immune response, it is essential to place these numerical results in biological context. Therefore, the PCA was combined with a confirmatory factor analysis (FAN) to elucidate the relevant pathways of the observed immune response. As a part of the FAN, the published literature was examined for descriptions of the similarities and differences in the known biological properties of the cytokines identified through PCA, in order to find common or complimentary patterns of function. Groupings of inflammatory mediator contributions to components one and two in the PCA were interpreted as latent factors and used as the basis for FAN. FAN seeks to model the observed variables as linear combinations of the provided potential factors. This form of analysis is based on regression modeling, and therefore provides additional weight to similar evidence found through PCA when there is similarity in the results.

While factor analysis is related to PCA, the two analyses are not identical. PCA takes a linear combination of the observed variables to derive synthesized latent variables; however, factor analysis takes the conceptually opposite approach. Using regression techniques, this method models the observed variables through linear combinations of potential latent variables that are provided. When the two methods produce models that are in agreement or similar, we interpret this as suggestive evidence that the latent variables are indeed playing an influential role in the observed pathology.

The literature-based analysis is a process by which we have utilized previously published studies that elucidate the mechanisms or behaviors of the signaling proteins under analysis. We extract the biological functions described as associated with the proteins and use those functions as labels in our feature transformation and factor analysis work. We believe that this is an efficient evidence-based method to describe what signaling proteins the transformation and factor analysis methods are emphasizing as important and putting them into a narrative that describes what role they are likely fulfilling in context.

## Results

As a paradigm of localized tissue injury, excisional biopsies were made on the lateral aspect of the thigh on Lewis rat hind limbs, as described in Section “Materials and Methods.” To elucidate changes in the immune signaling profile that occur at the cessation of heartbeat, cardiac arrest was induced as described in Section “Materials and Methods.” The testing of the second hypothesis stated in the introduction is through samples collected within 30 s of cardiac arrest, within a timeframe that is common in trauma-associated cardiac arrest events.

Samples taken from the wounded areas of skin and muscle were found to have significantly different levels and patterns of inflammatory mediator expression. These differences were evident across groups as well as across tissue.

**Figure 1:**
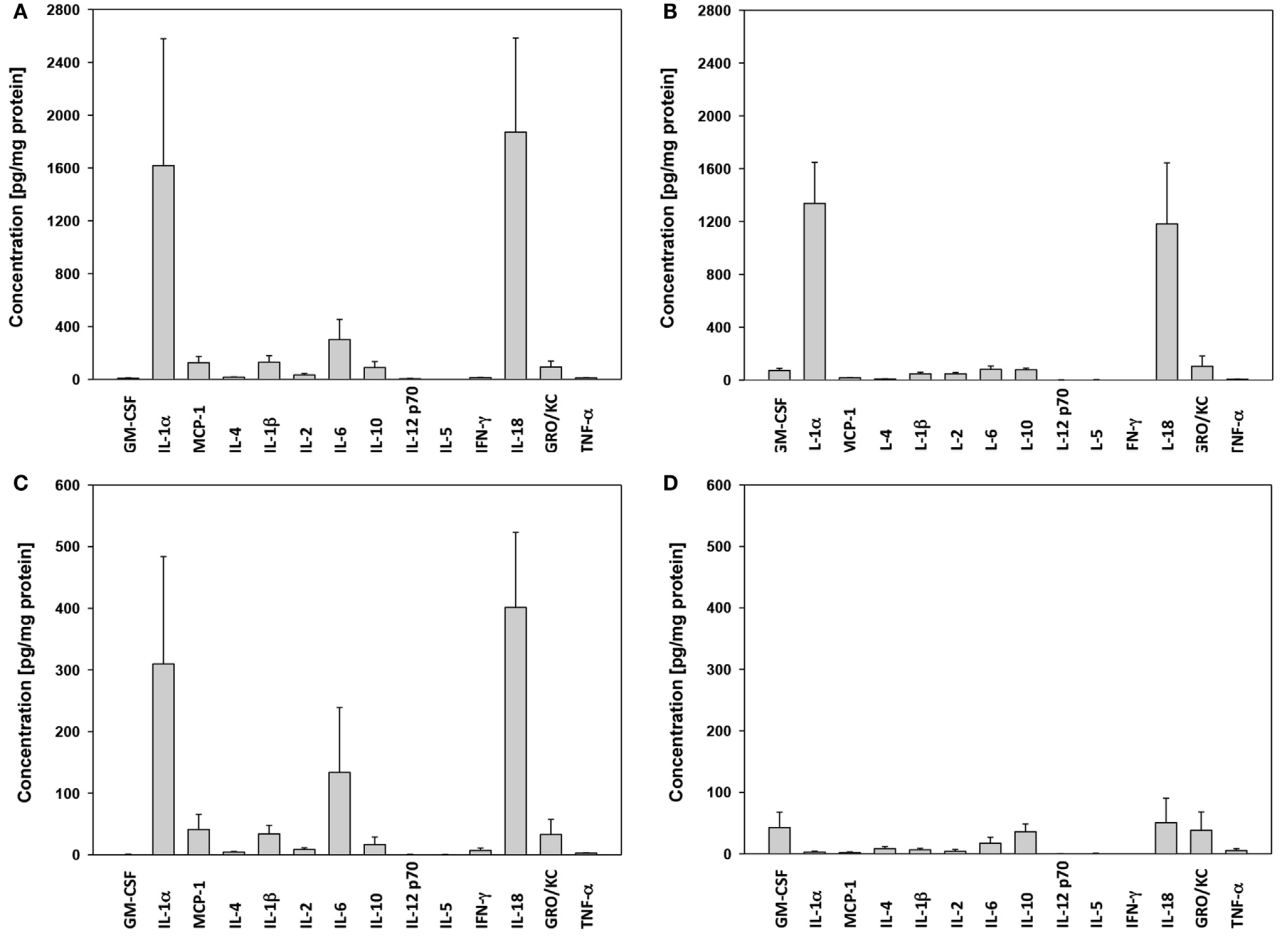
**Cytokine expression in skin and muscle tissue: “cardiac arrest group” vs. “wound group.”** The concentration of each cytokine in skin tissue in the “cardiac arrest group” **(A)** and the “wound group” **(B)** and in muscle tissue [“shock” **(C)** and the “wound group” **(D)**] across all time points, measured by Luminex™ as described in Section “Materials and Methods.” Cytokine concentrations are expressed in picogram per milligram total protein.

In skin, the levels of IL-18 and IL-1α present in “cardiac arrest group” animals (Figure 1A) were much higher than those seen in any other groups. MCP-1, GM-CSF, IL-10, IL-6, and IL-1β were all also elevated in the “injury group” animals (Figure 1B). In muscle, similar differences between “cardiac arrest group” animals (Figure 1C) and “injury group” (Figure 1D) were also seen, along with marked increases in IL-18, IL-1α, IL-6, IFNγ, and MCP-1.

**Figure 2:**
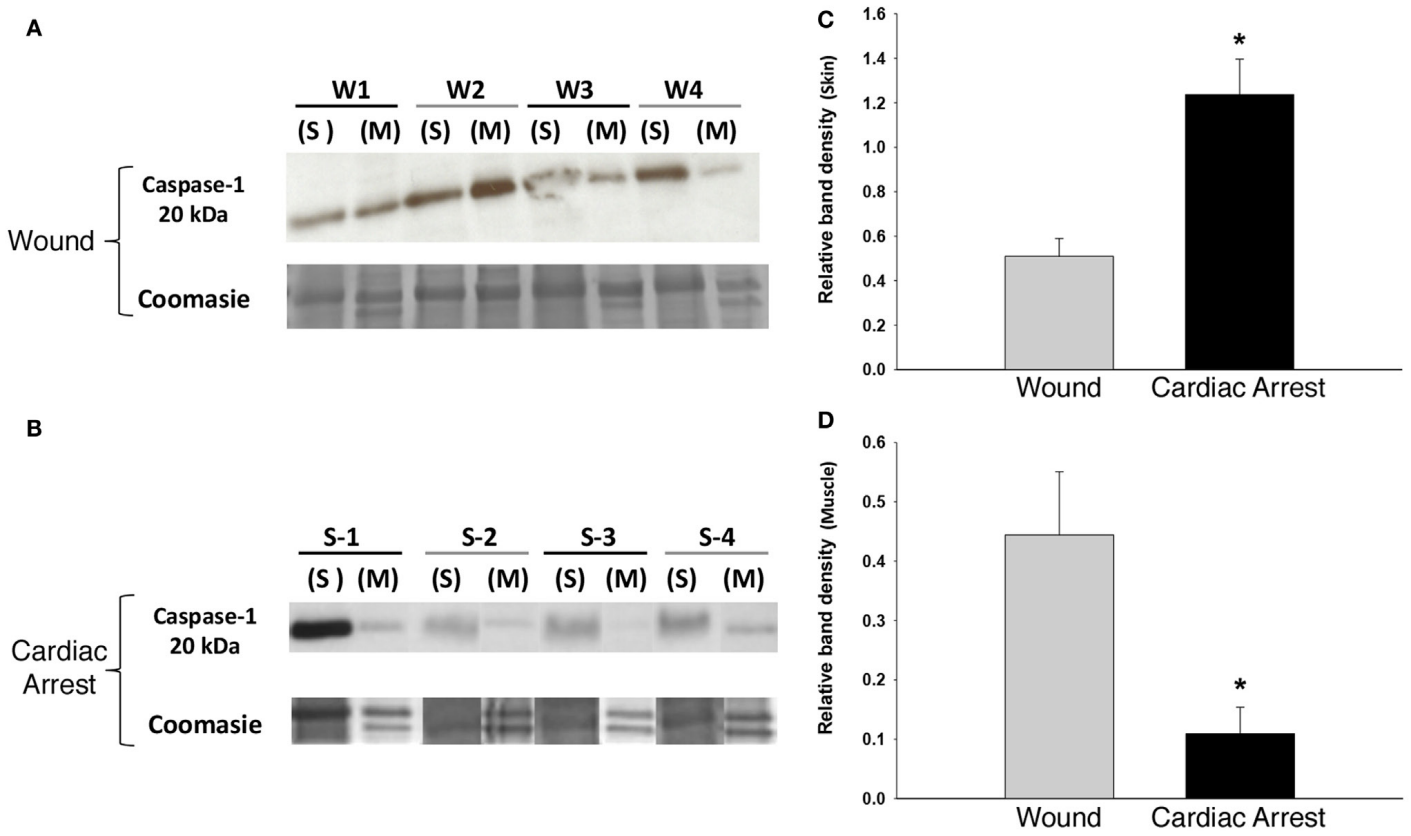
**Caspase-1 expression in skin and muscle tissue: “cardiac arrest group” vs. “wound group.”** Skin and muscle samples from both experimental groups (“cardiac arrest group” and “wound group”) were processed for protein isolation followed by Western blotting and analysis for active caspase-1 protein and Coomassie blue staining for loading control (see Materials and Methods). **(A,B)** Show a Western blot for caspase-1 in the “wound group” (W) and the “cardiac arrest group” (S), respectively. The numbers represent the sample from the individual animals in each group (*n*-4). Densitometric analysis of Western blots for active caspase-1 in skin **(C)** and muscle **(D)** (*P* < 0.05, as determined by one-way ANOVA).

The presence of IL-18 and IL-1β led us to hypothesize the presence and activation of the NLRP3 inflammasome in response to localized tissue injury and cardiac arrest. To test this hypothesis, we examined the expression of caspase-1, which is required for inflammasome activity (43). As determined by Western blotting analysis, caspase-1 was present in both skin and muscle in all study groups (Figures 2A,B). This finding, in combination with the presence of IL-18 and IL-1β, strongly indicates NLRP3 inflammasome activity. Similar levels of caspase-1 expression were found in both the skin (Figure 2C) and muscle (Figure 2D) of “injury group” animals, implying relatively equal levels of inflammasome activation. In contrast, “cardiac arrest group” animals expressed significantly more caspase-1 in skin than muscle, by approximately a factor of 12.

“Cardiac arrest group” skin was associated with elevated levels of caspase-1 and IL-18. In “cardiac arrest group” muscle, IL-18 levels were also markedly increased; however, caspase-1 levels were lower than in “injury group” muscle.

**Figure 3:**
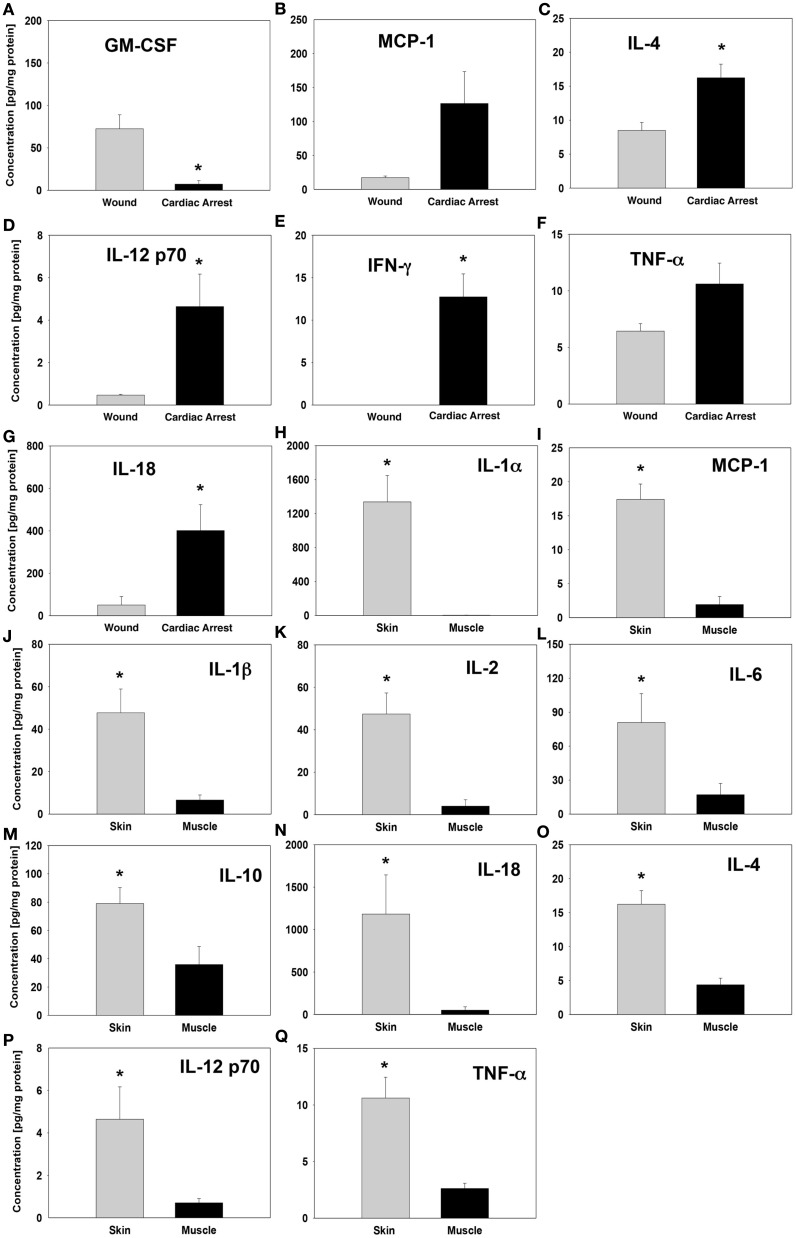
**Cytokine levels in “wound group” vs. “cardiac arrest group” and in skin vs. muscle tissue**. Tissue protein-normalized cytokine concentrations in “wound group” vs. “cardiac arrest group” **(A–G)** and in skin vs. muscle tissue **(H–Q)**. The concentrations of each cytokine were measured by Luminex™ as described in Section “Materials and Methods” and are expressed in picogram per milligram total protein. *P < 0.05 by one-tail heteroscedastic *t*-test.

Whereas significant inflammatory mediator concentration differences in the skin were observed between the “injury group” and “cardiac arrest group,” the levels were consistently higher in the “cardiac arrest group” animals (Figures 3B–G). The single exception was GM-CSF, which was present at a much higher concentration in the skin of “injury group” animals (Figure 3A). In “cardiac arrest group” animals, the cytokine with the highest differential elevation relative to “injury group” animals was IFNγ. The only cytokine elevated differentially between groups within muscle tissue was IL-18, whose expression was considerably higher in “cardiac arrest group” animals (Figure 3N).

Also notable was the contrast between skin and muscle tissue inflammatory mediator profiles, particularly within the “injury group” (Figures 3H–Q). Interestingly, the “injury group” animals displayed a much wider range of statistically significant differences in the expression of cytokines and chemokines between skin and muscle tissue as compared to “cardiac arrest group” animals. Although the concentration of many of these inflammatory mediators was actually higher in “cardiac arrest group” animals, the difference between skin and muscle tissue expression of each cytokine was much smaller. IL-4, IL-12p70, and TNFα were expressed at significantly different levels between skin and muscle tissues within the “cardiac arrest group” animals (Figures 3O–Q). These expression levels are also distinct from any inflammatory mediators within the “injury group,” revealing distinct immune signaling patterns in the two groups.

**Figure 4:**
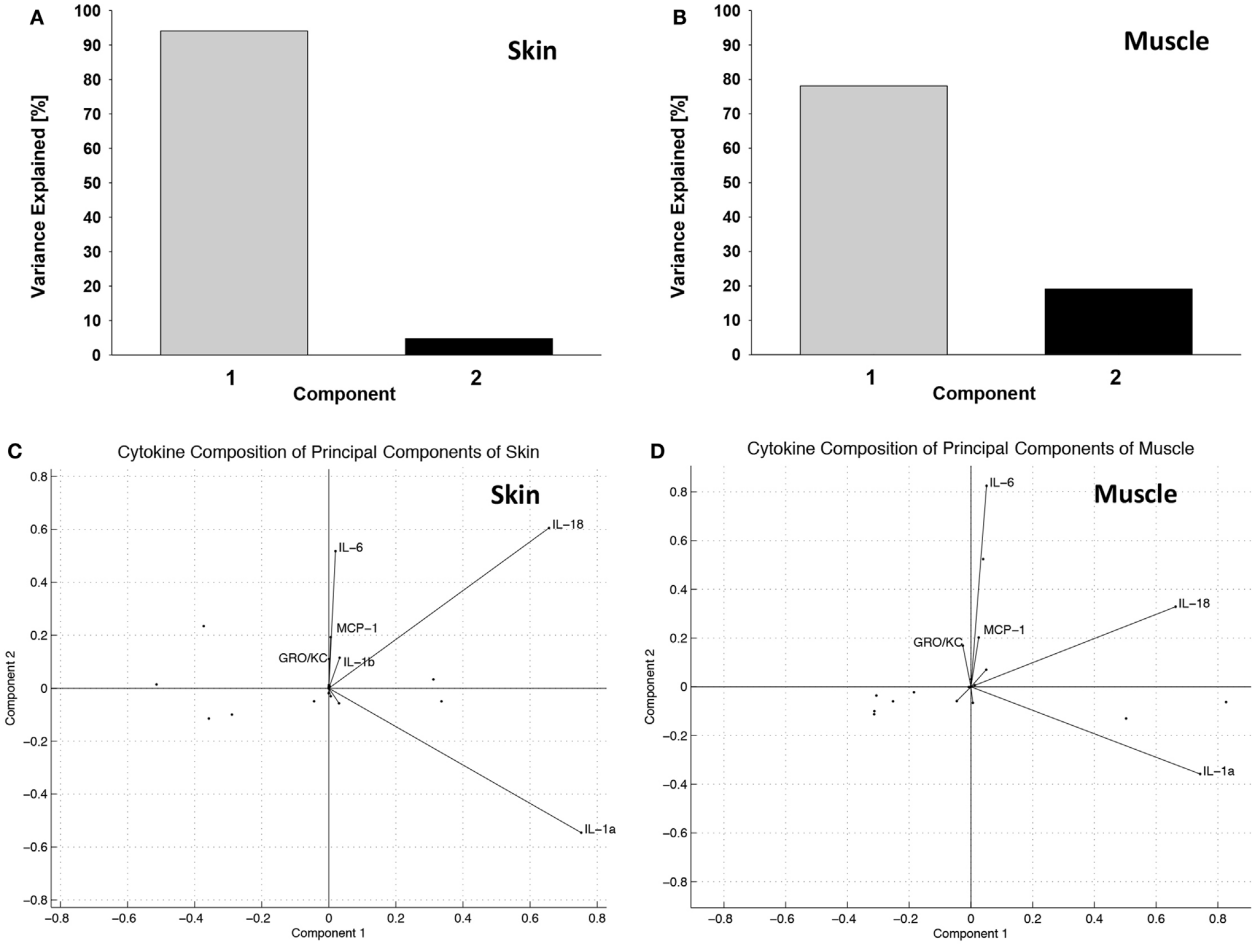
**Principal component analysis suggests distinct groups of inflammatory mediators induced in the “wound group” vs. the “cardiac arrest group” in skin and muscle**. **(A)** Percentage of variance explained by principal components in skin. Over 95% of the variance observed in skin can be explained by two latent variables, formed through linear combination of the original 14 variables (cytokines). Latent variables eliminate redundant information and identify the cytokines that are most informative about the observed immune response. **(B)** Percentage of variance explained by principal components in muscle. Over 95% of the variance observed in muscle can be explained by two latent variables, formed through linear combination of the original 14 variables (cytokines). **(C)** Principal component variable composition in skin. Each vector represents a cytokine’s contribution to each of the first and the second principal components. The lengths of the vectors indicate the strength of that cytokines contribution, and the direction indicates the proportion of principal component it is contributing to. This figure represents these relationships in the skin. **(D)** Principal component variable composition in muscle. This figure represents the strength of each measured cytokine’s contribution to each of the first and the second principal components, in muscle.

Principal component analysis synthesized two latent variables in skin (Figure 4A) and in muscle (Figure 4B). In skin, the first principal component was comprised mostly of IL-18 and IL-1α, while the second component was comprised of IL-18, IL-6, MCP-1, IL-1β, and GRO/KC (Figure 4C). The first principal component of muscle was also primarily made up of IL-18 and IL-1α, whereas the second principal component was mostly comprised of IL-6, IL-18, MCP-1, and GRO-KC (Figure 4D). TNFα and IL-4 were identified as important cytokines in both “cardiac arrest group” skin and muscle and are known to play a role in many immune regulation or intercellular signaling contexts (44–46). Although the contribution of each of these cytokines is similar in the first principal component, their contributions were substantially different in the second principal component. This finding suggests that the cytokines are complementary in some respects but have distinct roles in the inflammatory mediator network.

Principal component analysis suggested a potential role for GM-CSF in tissue injury (but not shock) skin. The converse was observed in the case of the chemokine MCP-1 (CCL2), which was highly expressed in the skin of “cardiac arrest group” animals, but expressed at far lower levels in the skin of the “injury group” animals. Furthermore, high levels of IFNγ were observed in the “cardiac arrest group” animals, while IFNγ was entirely absent from the “injury group” animals.

**Figure 5:**
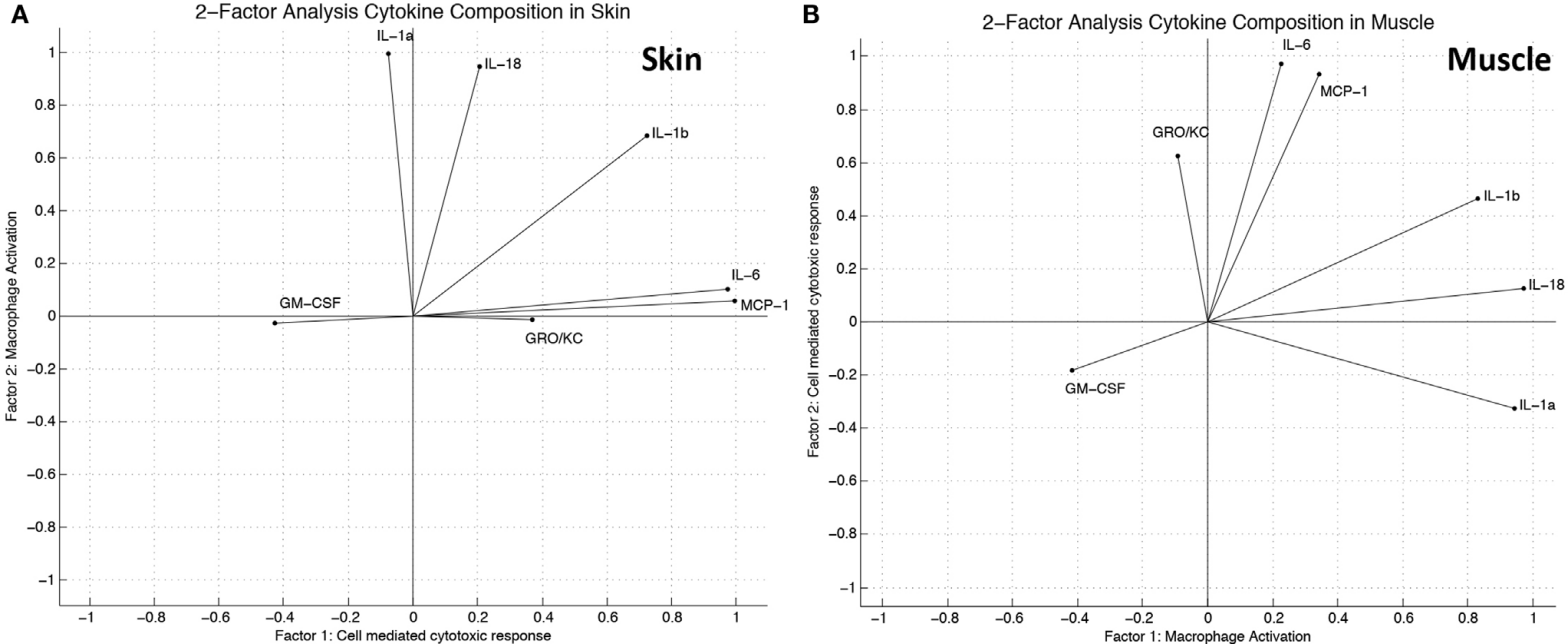
**Factor analysis suggests distinct groups of inflammatory mediators induced in the “wound group” vs. the “cardiac arrest group” in skin and muscle**. **(A)** Factor analysis (two factors) variable composition in skin. Modeled cytokine contribution to “cell-mediated cytotoxic response” and “macrophage activation” as calculated by linear combinations of provided latent factors. The direction of each vector indicates which factor the cytokine influences, while the length indicates the relative strength of influence as calculated by the model. **(B)** Factor analysis (two factors) variable composition in muscle. Modeled cytokine contribution to “macrophage activation” and “cell-mediated cytotoxic response” as calculated by linear combinations of provided latent factors.

Two-factor analysis of the skin generated a model of cytokine/chemokine contributions to the latent variables that was very similar to the representation derived by PCA (Figure 5A). Two-factor analysis of the muscle yielded a model that has similarities with the model derived by PCA; however, the overall agreement of the two models is not as strong as found in skin (Figure 5B). The functions of inflammatory mediators most heavily influencing each factor were correlated with descriptions of their biological function from the literature, and through this analysis labels for the latent variables, which were established as “macrophage activation” and “cell-mediated cytotoxic response.”

## Discussion

### Inferring Inflammasome-Driven Networks from Data-Driven Modeling

In the present study, we sought to elucidate immune/inflammatory signaling patterns in the response to skin and muscle damages, in the presence or absence of cardiac arrest. We reasoned that this would represent an important step in understanding the potential mechanisms that drive immunological responses to tissue damage at the earliest stages in the settings of complex injury.

In the present study, we hypothesized that application of high-dimensional feature transformation methods, combined with biological knowledge from reports in the literature, can provide a deeper understanding of the immune signaling processes that underlie injury-associated inflammation. Based on the multiplexed analysis of inflammation biomarkers coupled with data-driven modeling, we suggest that tissue injury leads to the differential induction of IL-1α and IL-18 in skin vs. muscle. This inflammatory profile is associated with elevated caspase-1 immunoblotting in skin but not muscle. Cardiac arrest greatly elevates IL-1α, IL-18, and caspase-1 in the skin. Thus, inflammasome activity appears to be central to the type and severity of the inflammatory response to local tissue injury, and this activity appears to be augmented greatly in the presence of cardiac arrest.

We utilized PCA in the current study, because this method is capable of quantifying the amount of information contributed by individual cytokines to the observed inflammation (25, 47, 48). Because this method evaluates the contribution of new information derived from each inflammatory mediator to each of the inflammatory processes observed, PCA assigns the highest scoring to cytokines most correlated with a specific inflammatory response. As utilized herein, PCA seeks to find the linear combination of the original 14 inflammatory mediators that captures the most variance in the smallest number of synthesized variables. Thus, since PCA can show which combination of inflammatory mediators lies in a particular principal component, this method can suggest inflammatory networks which interact together to drive a particular facet of the overall response.

Although a well-established numerical analysis method, additional evidence to support the interpretation of PCA results is desirable and can be provided by FAN (49–51). PCA and FAN are related methods and can intuitively be understood as providing confirmatory analysis. While PCA is based on variance, FAN takes as the input the number of hypothesized factors driving the observed process and then seeks to find the optimal linear coefficients for each cytokine to project from the hypothesized factors back to the original values measured for each cytokine. In short, PCA reduces the dimensionality of a given dataset, while FAN expands that dimensionality. In both cases, the effect is to expose the orthogonal aspects of the data in order to gain a better perspective of what measured parameters are most responsible for driving the observed process, which in the present study is inflammation. When the two methods produce coefficient scores for the inflammatory mediators that are similar to each other, this can be interpreted as evidence in support of the hypothesis that inflammation is, to some degree, driven by those particular mediators. However, if the two methods produce coefficient scores that do not agree, it is likely that the number of hypothesized factors is insufficient to capture the complexity of the inflammatory process.

Using such dimensionality reduction methods, we have previously inferred principal characteristics or drivers of inflammation in mice subjected to surgical trauma alone vs. that same trauma in combination with hemorrhagic shock (25). More recently, we have utilized PCA to suggest a key module in a multicompartment mechanistic mathematical model of inflammation and organ pathophysiology in endotoxemic swine (48), to suggest key physiologic effects of peritoneal suction as a therapy for sepsis (32), to connect *in vitro* and *in vivo* outcomes in the inflammatory response to implanted biomaterials (28), as well as suggesting key changes in metabolism that occur in the setting of pulsatile perfusion of livers prior to transplantation (29). Importantly, we have recently used these methods to suggest a role for the inflammasome (including elevated IL-18 and caspase-1) in a rat model of chronic neuropathic pain (42).

To better understand the observed immune signaling activity, we investigated two classes of insult: tissue injury only and tissue injury with shock. In the first model, the inflammatory response to surgically excised wounds in skin and muscle tissue without any secondary contaminants reveals immune signaling patterns primarily associated with wound healing. In the model of injury with shock, changes in the patterns of the inflammatory response to surgically incised wounds under conditions of cardiac arrest represent the disruptive effect of a disruption in blood flow and the associated stresses have on wound-healing signaling. Changes in immune signaling under these circumstances are caused by metabolic stress and other factors accompanying shock.

In the present study, we observed elevations in a wide range of proinflammatory mediators, including IL-1α, IL-1β, IL-4, IL-6, IL-12, IL-18, MCP-1, and TNFα. These networks of inflammatory mediators, observed in both the “injury group” and the “cardiac arrest group” are indicative of highly activated immune cells, such as dendritic cells, mast cells, and neutrophils. Dendritic cells are known as sensitive and potent pathogen presentation cells, but they are also becoming recognized as important in wound detection and healing in a variety of contexts (52–54). The specific role of these immune cells in a tissue is affected by the cytokine milieu present (23, 55). Importantly, most of these mediators have been implicated in the response to trauma/hemorrhage, in prior studies, especially TNFα (56–59) and MCP-1 (31, 35).

Although the specific milieu of the “injury group” and “cardiac arrest group” animals appeared distinct, PCA and FAN suggested a central role for IL-1α and IL-18, leading us to hypothesize and confirm the concomitantly elevated expression of activated caspase-1 as evidence for strong inflammasome activation in these tissues. As cells are killed, are damaged, or are induced into apoptosis by the wounding process, they release endogenous damage-associated molecular pattern (DAMP) molecules, such as uric acid crystals and high-mobility group protein B1 (HMGB1), molecules that trigger inflammatory response mechanisms (60–64). One DAMP-induced mechanism of particular relevance to the inflammatory signaling profile observed in this study involves the activation of the NLRP3 inflammasome (65), which is one of several multiprotein complexes that play important roles in inflammation and cell death (66). Deficiency or overactivation of these cytoplasm-based protein assemblies has been implicated in inflammation-associated damage in a variety of disorders (67), and inflammasome activation is widely regarded as critical in the initiation of the innate immune response. NLRP3 responds to DAMPs and involves a spontaneous protein assembly that forms primarily in the cytoplasm of macrophages, monocytes, some keratinocytes (65), and mast cells (68). The NLRP3 inflammasome is known to respond to cytokines as well as DAMPs including HMGB1 and uric acid crystals (69). The inflammasome, in turn, produces and secretes bioactive proinflammatory cytokines. This process initiates an inflammation and wound-healing cycle that may moderate and conclude successfully with functional wound healing or may lead to runaway inflammation resulting in dysfunctional wound healing (68).

Although all classes of inflammasome produce proinflammatory cytokines, each assembly only forms in response to specific stimuli. In the present study, we found evidence for the activation of NLRP3 (cryopyrin, NALP3) inflammasome. This inflammasome forms specifically in response to the types of DAMPs released in wounding and shock, as well as pathogen-associated molecular patterns (PAMPs) (70, 71). This class of inflammasomes leads to the recruitment of caspase-1 and the cleavage of pro-IL-1β and pro-IL-18 to yield the bioactive, proinflammatory cytokines IL-1β and IL-18. These cytokines are able to induce degranulation in polymorphonuclear neutrophil (PMN) leukocytes (72). Importantly, caspase-1 activity appears to be essential for the innate immune response, given that caspase-1-deficient mice are resistant to developing shock in response to PAMPs, such as LPS (73).

NLRP3-depleted mice produce neither IL-1β nor IL-18 but are still able to express IL-1α, IL-12p40, and TNFα (74). In the present study, we found large quantities of IL-1α in wounded skin across all groups, suggesting that the observed immune response may in fact be comprised of several components that are driven by different activation and signaling pathways. Furthermore, we observed larger quantities of IL-18 vs. IL-1β, in line with reported requirement for prestimulation, for example with LPS, for the detection of large quantities of IL-1β (73). These findings are also in line with our recent studies in a rat model of chronic constriction injury and neuropathic pain (42).

In addition to inflammasome-derived cytokines, analysis of the biological functions of the cytokines identified by PCA suggested other potential mechanisms of inflammation induced by skin injury and cardiac arrest. For example, GM-CSF was highly expressed in skin in the “injury group” animals, while this cytokine was relatively absent in the “cardiac arrest group” animals. Since GM-CSF is an important regulator of macrophage and granulocyte populations and has been shown to play an important role in the onset and propagation of inflammation (75, 76), its presence indicates strong macrophage recruitment and host defense or inflammatory activity. Thus, we hypothesize that GM-CSF plays a key role in the phenotype of the skin of the “injury group,” a role which we hypothesize is supported by cytokines, such as TNFα and IL-4. Numerous reports on the healing properties of GM-CSF in the literature support our PCA-derived hypothesis for the role of GM-CSF in the “injury group” (77–82).

In muscle, IL-6, IL-18, and IL-1α shared both direction and strength of influence in both PCA and FAN models of muscle. Interestingly, we have suggested that MCP-1 in some way controls IL-6 expression (31), and it was notable that MCP-1 figured in both PCA and FAN. Additionally, GM-CSF and IL-1β were influential variables in PCA but were not highly influential in FAN. These findings suggest that the muscle response to injury, both with and without shock, is likely driven by a larger number of latent factors than in skin.

The high concentrations of MCP-1 seen in the “cardiac arrest group” may possibly be caused by the cellular metabolic stress associated with cardiac arrest, resulting in insufficient cellular perfusion that would then lead to vasodilation, shock, and widespread degranulation of mast cells (83, 84). This chemokine helps recruit monocytes, dendritic cells, and memory T cells to sites of inflammation. Perhaps more significantly, MCP-1 exerts an important role in the degranulation of basophils and mast cells, facilitating the release of serine proteases, histamine, serotonin, and proteoglycans (70). In turn, mast cell and neutrophil degranulation induced by chemokines, such as MCP-1, and cytokines, such as IL-18, releases TNFα, eosinophil chemotactic factor, histamine, and a number of other factors (85, 86). This release alerts other nearby cells of injury and is a part of the cascade that begins the immune response to the injury. This hypothesis is supported by evidence that the mast cells are required for optimal migration of dendritic cells, swelling, neutrophil infiltration, and other effects associated with wound healing (87). We have recently suggested, through combined *in vitro*, *in silico*, and clinical studies that liver-derived MCP-1 is a central mediator in dynamic networks of inflammation in trauma (31).

In the “cardiac arrest group,” it is likely that the release of TNFα as a proapoptotic cytokine was triggered, leading to higher concentrations of TNFα in the skin of the “cardiac arrest group.” However, the role of TNFα is varied and dependent on a wide range of factors, including receptor binding (TNF-R1 vs. TNF-R2), the local cytokine milieu, the presence of reactive oxygen species, as well as many additional factors (88, 89). It is therefore not surprising that TNFα is seen also seen in the “injury group” although not in as primary a role as in the “cardiac arrest group.”

TNFα also plays a potentially important role as a costimulator of IFNγ production with IL-12 (IL-12p70) (90–92). Levels of IFNγ in the skin of the “cardiac arrest group” suggest this as an important role of IL-12 in this context but is also capable of a variety of immunological capabilities, including stimulating proliferation in resting peripheral cells, promoting the generation of lymphokine-activated killer cells (LAK cells), and augmenting the cytolytic activity of natural killer cells (NK cells) (93–96).

Another prominent cytokine observed in our studies was IL-4, which is known to stimulate IgE B cell differentiation as well as alternative activation of macrophages into M2 repair cells (97), suggesting that IL-4 may be supporting a wound-healing pattern in the “injury group.” However, in the “cardiac arrest group,” significantly higher concentrations of IL-4, as well as the increased presence of additional cytokines that are associated with shock, suggest that in this context IL-4 may be acting as a promoter of IgE synthesis, contributing to the shock reaction and promoting widespread rapid degranulation of PMN leukocytes. Importantly, in the “injury group,” high levels of GM-CSF are seen along with moderate amounts of IL-4. This same combination of cytokines has been shown to enable the maturation of monocytes into dendritic cells *in vitro* (98).

### Limitations

There are several challenges and limitations in this study. This study focuses on the early inflammatory phase following injury and therefore does not follow wounds through full healing. Although statistically significant differences in the expression levels of multiple inflammatory mediators were found between groups, the study size was limited to eight animals (four in the “injury group” and four in the “cardiac arrest group”). Additionally, a single time point proximal to the time of injury was studied. Future studies would ideally extend the findings reported here with larger study groups, sampled at multiple longitudinal time points. Our focus on the inflammasome was derived from an analysis of a limited number of inflammatory mediators. Thus, it is possible that (1) others, unmeasured inflammatory mediators play key roles in postinjury inflammation and (2) that our analysis methods were insufficient to inform our hypotheses. With regard to the former, we attempted to focus our analysis on mediators that have been studied previously in wound biology. With regard to the latter, we attempted to use corroborative methodologies (PCA and FAN) to gain confidence in our conclusions. In skin, the agreement between PCA and FAN models was high across all measured mediators, providing additional evidence that two factors are explanatory. Nonetheless, confirmation of this hypothesis requires studies in which the inflammasome, caspase-1, IL-1α, and/or IL-18 is antagonized.

## Conclusion

The inflammasome appears to govern the early inflammatory response to tissue injury in the skin, releasing proinflammatory factors likely to initiate host defense and clearance of debris. Normal wound healing is characterized and regulated by characteristic immune signaling patterns, reflected in characteristic cytokine and chemokine profiles of skin and muscle (17, 99–102). The mediator profile characteristic of an early inflammatory response to tissue injury is modified extensively as a result of cellular stress induced by shock, likely interfering with eventual wound repair and regeneration. Moreover, the inflammatory network characteristics of skin and muscle injury responses (both with and without severe shock) are recognized as distinct from each other, as inferred from data-driven computational analyses. These findings support the use of feature transformation methods, in combination with literature-based analysis of the underlying biological functions, as a method to separate relevant biological processes from irrelevant homeostatic processes, or other background functions.

## Conflict of Interest Statement

The authors declare that the research was conducted in the absence of any commercial or financial relationships that could be construed as a potential conflict of interest.

## Abbreviations

BRD, band relative density; CTA, composite tissue allotransplantation; *df*, degrees of freedom; *F*, *F*-statistic (ratio of the between-group and within-group mean squares); FAN, confirmatory factor analysis; “injury only,” excisional wound group; LPS, lipopolysaccharide; MS, mean squares (the ratio SS/*df*); NMR, nuclear magnetic resonance; PCA, principal component analysis; PMN, polymorphonuclear neutrophil; “shock associated with cardiac arrest,” cardiac arrest group; SS, sum of squares.
